# A comprehensive database of exosome molecular biomarkers and disease-gene associations

**DOI:** 10.1038/s41597-024-03015-7

**Published:** 2024-02-15

**Authors:** Yue Qi, Rongji Xu, Chengxin Song, Ming Hao, Yue Gao, Mengyu Xin, Qian Liu, Hongyan Chen, Xiaoting Wu, Rui Sun, Yuanfu Zhang, Danni He, Yifan Dai, Congcong Kong, Shangwei Ning, Qiuyan Guo, Guangmei Zhang, Peng Wang

**Affiliations:** 1 Department of Gynecology of the First Affiliated Hospital, Harbin Medical University, Harbin, 150081 China; 2 College of Bioinformatics Science and Technology, Harbin Medical University, Harbin, 150081 China; 3 Department of Colorectal Surgery, Harbin Medical University Cancer Hospital, Harbin, 150081 China

**Keywords:** Cancer, Molecular biology

## Abstract

Exosomes play a crucial role in intercellular communication and can be used as biomarkers for diagnostic and therapeutic clinical applications. However, systematic studies in cancer-associated exosomal nucleic acids remain a big challenge. Here, we developed ExMdb, a comprehensive database of exosomal nucleic acid biomarkers and disease-gene associations curated from published literature and high-throughput datasets. We performed a comprehensive curation of exosome properties including 4,586 experimentally supported gene-disease associations, 13,768 diagnostic and therapeutic biomarkers, and 312,049 nucleic acid subcellular locations. To characterize expression variation of exosomal molecules and identify causal factors of complex diseases, we have also collected 164 high-throughput datasets, including bulk and single-cell RNA sequencing (scRNA-seq) data. Based on these datasets, we performed various bioinformatics and statistical analyses to support our conclusions and advance our knowledge of exosome biology. Collectively, our dataset will serve as an essential resource for investigating the regulatory mechanisms of complex diseases and improving the development of diagnostic and therapeutic biomarkers.

## Background & Summary

Exosomes (30–150 nm size) are cell-derived vesicles containing proteins, nucleic acids and other metabolites that are widely found in various body fluids such as plasma, cerebrospinal fluid, urine, and saliva^1,2^. Emerging evidence suggests that exosomes can play essential roles in intercellular communication and act as functional mediators in many serious diseases and the progression of various cancers^3,4^. Detecting the molecular features in exosomes can expand our knowledge of causative signal transduction behind complex disease pathology and improve the development of diagnostic and therapeutic biomarkers^5^. With the development of sequencing technology, several RNA molecules, such as microRNAs (miRNAs)^6^, mRNAs^7^, long non-coding RNAs (lncRNAs)^8^, and circular RNAs (circRNAs)^9^ have been found in exosomes and play equally critical regulatory roles. For example, lncRNA BCRT1 can be transferred to macrophages via exosomes, thereby promoting phagocytic M2 polarization and enhancing its effect on tumor progression^10^; circSATB2, which has high sensitivity and specificity for clinical detection is highly expressed in the serum exosomes of lung cancer patients and is associated with lung cancer metastasis^11^; The discovery of mRNA CA9 enrichment in the urine of bladder cancer patients validates the potential of urinary exosomal CA9 mRNA as a new liquid biopsy method for the diagnosis of bladder cancer^12^. Thus, we specifically focused on nucleic acid biomarkers and collected experimentally validated exosomal RNAs that will help researchers improve the accuracy of early cancer screening and provide new insights into the mechanisms of cancer development.

With the increase in the number of high-throughput sequencing data of human body fluid, more and more molecular regulatory roles of exosomal RNAs have been revealed^13^. Several databases have been established to store disease-related human body fluid exosomal RNAs, such as exoRbase^14^, exRNA Atla^15^, ExoCarta^16^, etc. These databases have not only contributed significantly to the study of the biological function of exosomal RNA but have also documented in detail the relationship between exosome RNA, protein, and lipid. However, continuously updated experimentally confirmed disease-associated exosomal RNAs are not included in these databases. Apart from that, previous work focused on bulk data and ignored the altered regulatory relationships that may occur in single-cell data sets. To fill these gaps, we have developed ExMdb, a comprehensive database of exosomal nucleic acids biomarkers and disease-gene associations from published literature and high-throughput datasets.

**Fig. 1:**
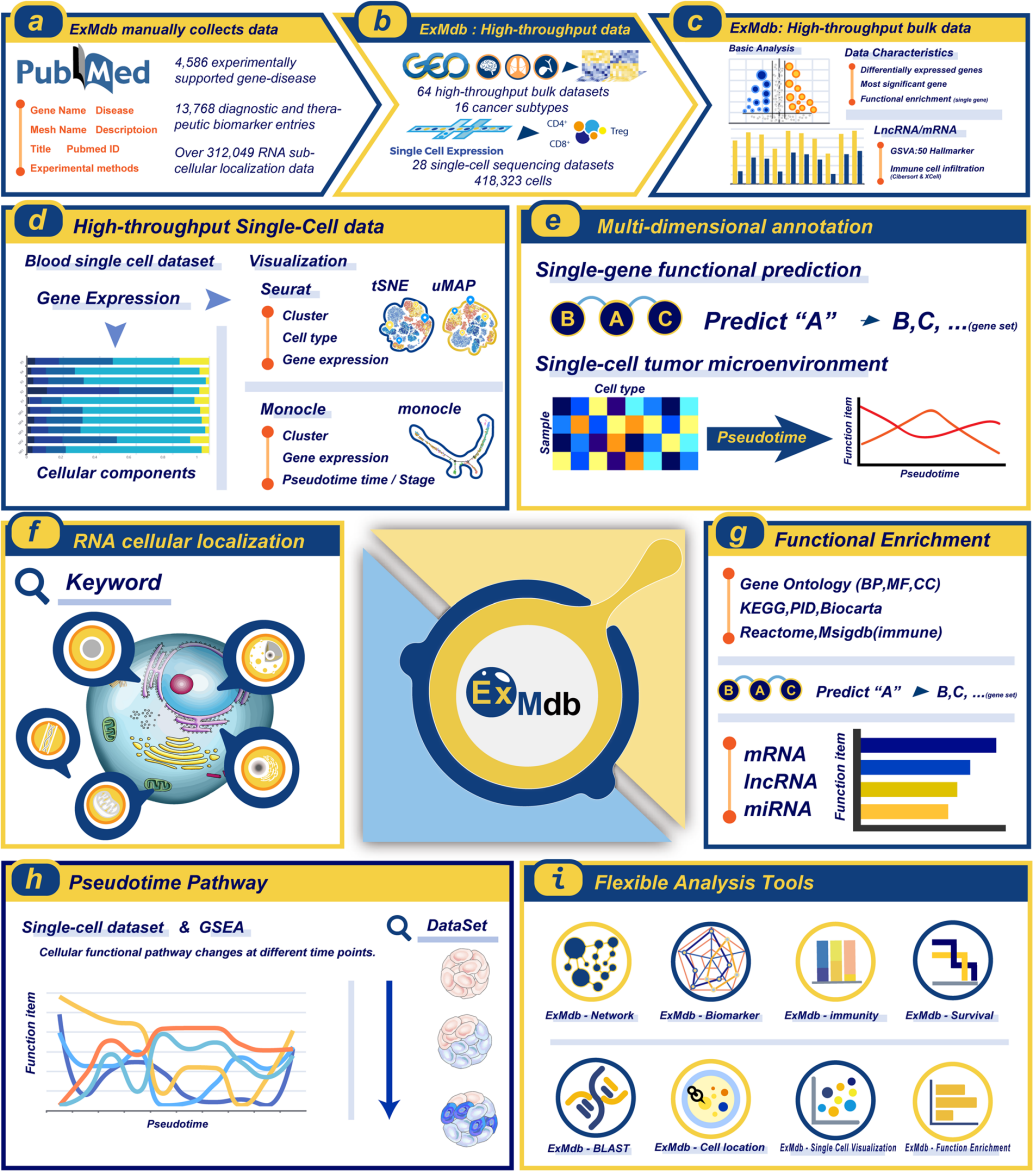
Overview of exosomal data content and functionality. (**a**) Overview of experimental validation data acquisition. (**b**) Overview of bulk data and scRNA-seq data information. (**c**) Bulk data processing and analysis pipeline. (**d**) ScRNA-seq data processing and analysis pipeline. (**e**) Data prediction and multi-level annotation. (**f**) Plots of RNA subcellular localisation results. (**g**) Multi-context functional annotation. (**h**) Overview of pseudotime analysis results. (**i**) A panel of data analysis tools.

We focused on the collection of exosomal nucleic acid features and disease-gene relationships for hundreds of diseases, including (i) more than 4,000 experimentally supported gene-disease associations in exosomes and blood; (ii) more than 13,000 diagnostic and therapeutic biomarker entries, which contain multiple types of RNA (including mRNA, lncRNA, miRNA, circRNA, etc.); (iii) identification of causative exosome molecules from 64 high-throughput bulk and 28 scRNA-seq datasets; (iv) detailed information of RNA subcellular locations by manual curation from literature and related data sources. In addition, this study provides multi-omics data, (e.g. functional omics information, clinical annotation information, RNA interactions information, etc.). Our dataset will serve as an essential resource for investigating causative signal transduction behind complex disease pathology and finding reliable diagnostic biomarkers. A schematic overview of this study design is shown in Fig. 1.

## Methods

We collected data from public databases containing curated, inferred, literature-based information to create a database for collecting exosome molecular information. With curated disease genes, phenotypes, and cancer-associated exosomal RNAs, this work tries to provide researchers with reliable assistance by establishing an extensive, high-confidence public resource for investigating the regulation mechanism of complex diseases and improving the development of diagnostic and therapeutic biomarkers.

### Experimentally validated gene-disease associations

We collected high-confidence records from published literature. We searched the PubMed database using the following keywords: “Exosome lncRNA Cancer”, “Exosome mRNA Cancer”, “Exosome CircRNA Cancer”, “Exosome mRNA Cancer”, “Exosome Non-Coding Cancer”, and downloaded all relevant literature containing cancer-associated exosomal RNA as cancer biomarkers. A total of 4,937 references were collected. Next, we extracted valid information and collected items in the literature by manual screening. During the manual screening phase, the selection criteria for article entries revolved around the presence of RNA in exosomes and whether this RNA plays a regulatory role in the onset and progression of cancer. We manually extracted experimentally supported associations between RNA and cancer from published papers, with all selected studies being reviewed by at least two researchers. The exosomal RNA-disease associations were verified using high-confidence experimental methods, including qRT-PCR, Western blot, dual-luciferase reporter, *in vitro* knockdown, ChIP-Seq, RNA immunoprecipitation, mass spectrometry, immunofluorescence, CRISPR/Cas9, flow cytometry, ELISA, and cell invasion. In this step, we collected gene symbol, RNA type, disease name, species, article title, PubMed ID, experimental validation method, and a brief functional description of the exosomal molecule. At the disease level, we standardized the disease names by MeSH to facilitate user queries. We collected 4,586 exosome RNA-disease associations from *Human* and *Mouse* from 159 diseases. Different RNA types, such as miRNAs, lncRNAs, mRNAs, and circRNAs, have also been collected. Finally, 10,084 experimentally supported biomarkers were manually curated from the literature.

### Experimentally validated biomarkers

We performed manual curation of experimentally supported biomarkers to provide insight into cancer diagnosis and therapy. A gene was selected as a biomarker based on its association with drug resistance processes, circulation, survival, immunity, metastasis, recurrence, cell growth, epithelial-mesenchymal transition (EMT), apoptosis and autophagy, validated by qRT-PCR, Luciferase reporter assay, Western blot, overexpression, ChIP, cell proliferation assay, RIP, ELISA, CRISPR/Cas9, RACE, siRNA transfection, immunoblotting, RNA knockdown. Given the diversity of RNA molecules linked to cancer, we have manually collated experimentally confirmed RNAs to provide new insights into tumor diagnosis and treatment. Our dataset currently contains 13,768 experimentally validated hallmark RNA molecule entries.

### High-throughput sequencing data

To explore the expression variation of exosomal RNAs and identify risk factors, we collected 90 high-throughput datasets, including 63 bulk and 27 scRNA-seq data. The high-throughput expression profiles were derived from the Gene Expression Omnibus (GEO)^17^ and exoRbase 2.0^14^. Exosomal genes were mapped to annotation files derived from GENCODE (release 41, GRCh38)^18^ to identify different types of genes. Expression profiles with more than 10 samples and 2000 genes were collected for each bulk dataset. For each scRNA-seq dataset, samples with more than 100 cells were retained. These datasets include common human diseases such as breast cancer, lung cancer, and coronavirus disease 2019 (COVID-19).

### Subcellular localization data

We collected a total of 312,049 RNA subcellular localization data covering 27,785 RNAs in several databases (e.g., lncATLAS^19^, exoRBase^14^, CSCD^20^) and published literature. We manually review the literature for explicitly mentioned cellular localization information and document the tissue, location, RNA name, and PubMed ID of the literature. For each RNA subcellular localization record, we listed a statistical table indicating the number of collections supporting the current dataset.

### Functional data

To provide comprehensive contexts for functional annotation of exosomal RNAs, we collected functional gene sets of Gene Ontology (GO), biological pathways, and cancer hallmark processes. For GO annotation^21^, we collected gene sets of 7,658 biological processes (BP), 1,738 molecular functions (MF) and 1,006 cellular components (CC). A total of 2,982 biological pathway gene sets from the Kyoto Encyclopedia of Genes and Genomes (KEGG), BioCarta, Reactome, and other biological pathway databases were collected from MSigDB^22^. We also collected 50 hallmark gene sets^22^ representing specific, well-defined biological processes. We performed a hypergeometric test at the gene level to evaluate the enrichment significance based on different functional contexts. At the single-cell level, the gene set variation analysis (GSVA)^23^ method was used to evaluate the cellular functional activation status and states in each dataset.

### Clinical data

We collected 74 high-throughput expression profiling data and corresponding clinical information from The Cancer Genome Atlas (TCGA, https://portal.gdc.cancer.gov/), and Gene Expression Omnibus (GEO)^17^. We applied the following selection criteria to filter the data: (i) The dataset contains sample prognostic information; (ii) The dataset contains a sufficiently large sample size (N > 30); (iii) The probes on the platform are annotated with public identifiers (e.g. Gene Symbol, GenBank, UniGene ID, etc.). The series matrix files were collected from the GEO dataset. In the data pre-processing, we used log2 transformation to reduce the effect of outliers and make the data more consistent with a normal distribution for further analysis.

### Statistical analysis

We performed gene expression differential analysis using the Limma package (v3.58.0). Survival analysis results were calculated using the survival package. In the immunology module, we separately used the Cibersort (v1.0) and XCell (v1.1.0) packages to calculate cell proportions in the samples. For the visualisation of single-cell data, we used the Seurat (v4) for cell clustering and the Monocle (v2) to compute pseudotime information of the cells. We performed large-scale analyses using a number of machine learning algorithms including random survival forest (RSF), Lasso, stepwise Cox, CoxBoost, elastic network (Enet), Ridge, partial least squares regression for Cox (plsRcox), supervised principal components (SuperPC), and survival support vector machine (survival-SVM). For each dataset, we screened for prognostic genes using univariate COX regression analysis. We randomly selected 2/3 of the samples as the training cohort and the remaining 1/3 as the validating cohort. We then integrated these machine learning algorithms to construct a robust prognostic prediction model. The C-index and AUC values were used to evaluate the performance of each model^24,25^.

## Data Records

All the data files are stored in the Figshare^26^ and are available under the terms of CC-BY 4.0.

The dataset contains a total of 6 data sets, covering the underlying data for the data composition and visualization tools. A brief description of all the data follows:

(i) The Experimental validated gene-disease.csv file contains the experimentally validated cancer-exosome RNA relationships recorded including gene name, species information, disease name, cell line/tissue information used for the experiment and experimental methods.
(ii) The Experimentally validated biomarkers.csv file contains the cancer-related lncRNA biomarkers supported by the experimental literature. The data include the lncRNA name, disease name, experimental method, information on the sample used in the experiment, PubMed ID and the regulatory direction of the RNA in this experimental sample, the biological processes associated with these lncRNA biomarkers (including drug resistance, circulation, survival, immunity, metastasis, recurrence, cell growth, EMT, apoptosis and autophagy).
(iii) Cell locations.csv file contains RNA subcellular localization data, which includes RNA Ensembl ID, subcellular localization, tissue, experimental method, data source and Gene Symbol/Gene ID.
(iv) High-throughput annotation.csv file provides detailed annotation information for all high-throughput datasets, including GEO accessions, cancer type, number of samples, data type, and platform Information.
(v) The clinical annotation information.csv data contains the abbreviation of the cancer name, cancer type, the number of samples and the data source for the relevant data.
(vi) Functional data.rar data provides all the functionally annotated gene sets, including GO-BP/MF/CC, hallmark gene set and immune gene set in MSigDB.

## Technical Validation

To verify and validate the accuracy of the experimentally supported exosomal biomarkers and disease-gene associations, the data extraction process was independently performed by the authors and subsequently cross-checked. Disagreements regarding data extraction were resolved by consensus. Information retrieval was performed manually. The exosomal data contains 4,586 gene-disease associations, 13,768 diagnostic and therapeutic biomarkers, and 312,049 nucleic acid subcellular locations. We proofread and validated these data by pulling high-confidence experiments from articles such as qRT-PCR, Western blot, dual-luciferase reporter and CRISPR/Cas9. In the quality control, we standardized the disease names by MeSH and unified the gene symbols and Ensembl IDs of all the exosomal genes.

To ensure that the results are statistically significant, comparative analysis is always performed on at least 6 samples (3 untreated vs 3 treated). As more samples will better avoid biological bias and make the results more accurate, we used 10 samples as a stricter criterion to filter the bulk expression profiles. A number of studies have designed and performed comparative analyses in at least 10 samples (5 untreated vs 5 treated) to identify exosome biomarkers. For example, a recent study performed comparative gene expression profiling analysis of serum-derived exosomal miRNAs from 5 patients with small cell lung cancer and 5 controls with lung nodules^27^. To further ensure the reliability of the experimental results, a sufficient number of genes should be provided for large-scale bioinformatic analysis. As a quality control, we filtered the expression data with at least 2,000 genes. For example, a recent study performed in silico identification of lncRNA-miRNA gene networks of acute kidney injury based on at least 2,000 genes with significant changes in expression^28^. As low cell numbers may not reflect the heterogeneity of different cell types, we used a minimum of 100 cells as a quality control for filtering the scRNA-seq dataset. In a previous study, false discoveries of differential expression in single-cells were systematically confronted and the degree of heterogeneity between replicates varied in at least 100 cells^29^. These criteria were also used in our previous studies^30,31^.

**Fig. 2:**
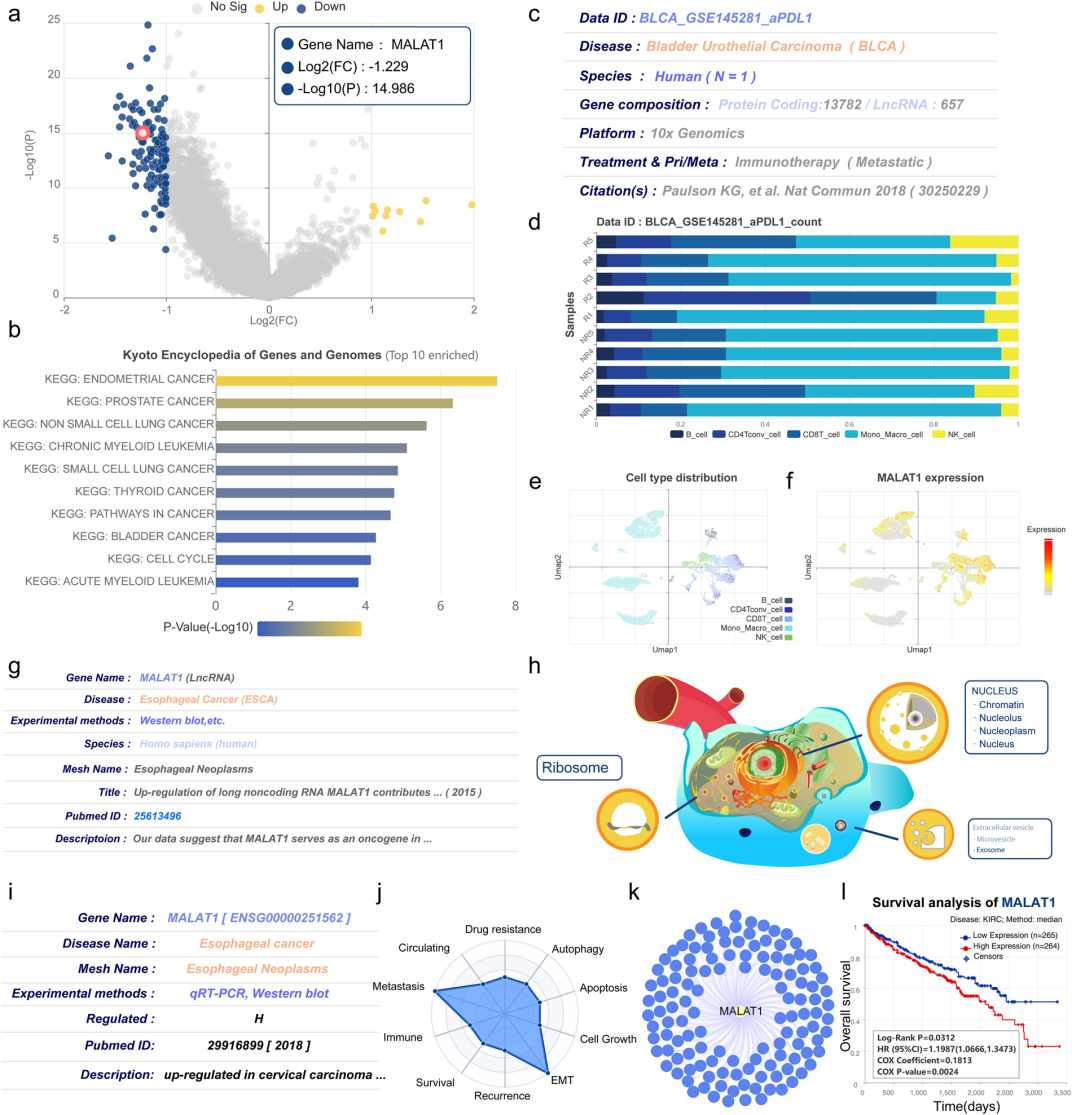
Examples of technical validation of the data. (**a,b**) Differential expression and functional enrichment analysis of MALAT1 in the bulk dataset. (**c**) MALAT1 annotations in scRNA-seq data. (**d**) The proportion of cells in different samples. (**e,f**) Cell type annotation and MALAT1 expression in different cell clusters. (**g,h**) Recorded entries of MALAT1 in the experimental validation dataset and its subcellular localization. (**i,j**) Cancer biomarker annotations of MALAT1. (**k,l**) The interaction network and survival analysis of MALAT1.

To further check the quality of the data, we validated the reliability of relevant exosomal gene records in the context of fluctuating expression and their reported biological function in a variety of diseases. We took the example of the gene MALAT1, which has been extensively studied and shown in previous studies to play a key regulatory role in several cancers^32^ (Fig. 2). Based on the high-throughput microarray sequencing dataset, we found that MALAT1 was significantly differentially expressed in the GSE133684^33^ (PDAC) dataset (p < 0.001, Fig. 2a). In the functional enrichment results, MALAT1 was found to be enriched in a large number of cancer-related pathways and plays an important role in tumourigenesis (Fig. 2b). Although dispensable for normal physiology, MALAT1 is important for the regulation of cancer-related pathways regulation^34^ and is associated with several cancer signalling pathways including MAPK/ERK^35^, PI3K/AKT^36^, β-catenin/Wnt^37^, Hippo-YAP^38^, VEGF^39^, etc. We also checked the quality of exosome/body fluid-related scRNA-seq datasets. Disease information, affected organs, number of samples, species and citation information were collected to provide more comprehensive and accurate information (Fig. 2c). For example, in the immunotherapy dataset GSE145281^40^, the proportional distribution of immune cells was significantly different between samples, reflecting individual differences in the tumour immune microenvironment under immune checkpoint blockade (Fig. 2d). Cell clusters and immune cell distribution are shown in Fig. 2e. We found that MALAT1 was expressed at relatively high levels in CD8 + T cells (Fig. 2f). These findings were consistent with a previous study that identified an important functional role for MALAT1 in the regulation of CD8 + T cell differentiation using a functional gene knockdown screen experiment^41^. Similarly, in the experimental dataset, there are several records of MALAT1 regulatory relationships validated in cancer. For example, Hu *et al*. identified MALAT1 as an oncogene in ESCC that regulates ESCC growth through alteration of the ATM-CHK2 signalling pathway^42^. For each entry, we provided a brief functional description of the RNA’s regulatory role in cancer progression and links to related articles for quick access (Fig. 2g). We collected extensive RNA annotation information in subcellular localization maps showing that MALAT1 is more abundant in the nucleus. This is also supported by several studies^2,43^. RNA localization and data source information is available in our subcellular localization dataset (Fig. 2h). For the quality control of each RNA localization, we have provided a statistical table showing the number of collections supporting the current dataset. For further understanding of RNA functions in cancer, we collected information on RNA abundance and regulation in the 10 classical cancer-related pathways (Fig. 2i,j). Based on experimental validation, we found that MALAT1 was associated with metastasis and EMT progress in esophageal cancer (EC). A recent study showed that up-regulation of MALAT1 contributes to proliferation and metastasis in EC^42^. Furthermore, MALAT1 could promote EMT and metastasis of EC cells through the Ezh2-Notch1 signaling pathway^44^.

In addition, we have integrated RNA interactions and clinical prognostic information to check the prognostic power of the data. We found that MALAT1 is able to regulate multiple RNAs and can be used as a prognostic marker for a variety of cancers, as shown in Fig. 2k,l. Through competing endogenous RNA (ceRNA) interaction, MALAT1 acts as miRNA “sponges” and dynamically regulates the expression of downstream targets (Fig. 2k). These types of ceRNA interactions have also been captured and characterized in previous studies^30,45,46^. For example, MALAT1 acts as a ceRNA for miR-23b-3p and attenuates the inhibitory effect of miR-23b-3p on ATG12, leading to chemo-induced autophagy and chemo-resistance in gastric cancer^47^. In addition, we found that up-regulation of MALAT1 confers poor prognosis in the TGGA-KIRC dataset (Fig. 2l). MALAT1 promotes progression of clear cell kidney carcinoma (KIRC) by regulation of miR-194-5p/ACVR2B signaling^48^. The results showed that highly expressed MALAT1, and lowly expressed miR-194-5p were associated with larger tumor size, advanced TNM stage and poor prognosis of KIRC patients.

**Fig. 3:**
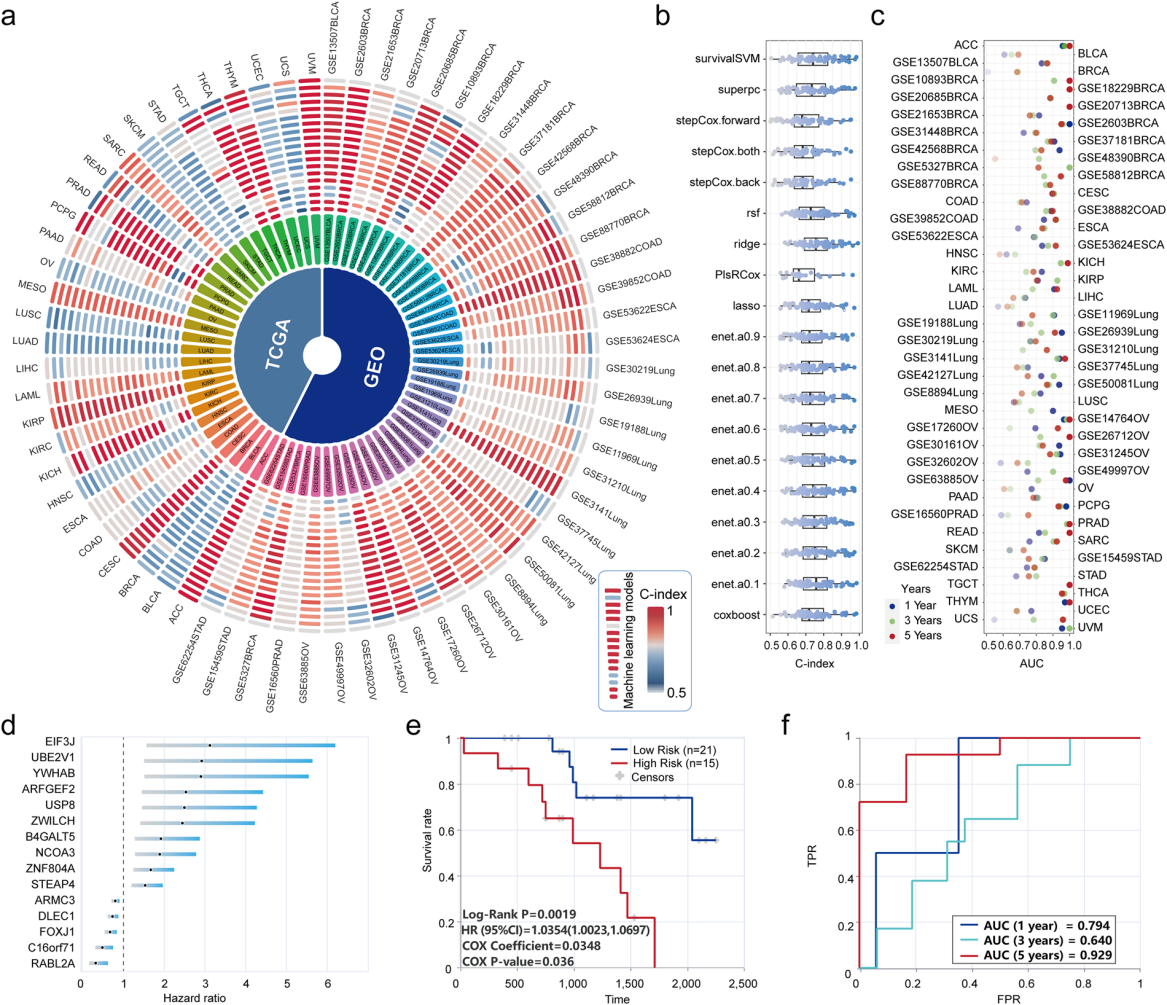
Performance of machine learning models for biomarker identification. (**a**) Landscape of machine learning model results for each dataset. (**b,c**) The distribution of C-index and AUC values across different models and datasets. (**d**) Several exosomal genes were significantly associated with ovarian cancer prognosis. (**e**) Survival curves of the validation cohort based on prognostic exosomal genes. (**f**) ROC curves of prognostic exosomal genes for 1, 3 and 5-year survival.

To further validate the reliability of our dataset, we performed large-scale analyses using a set of machine learning algorithms (Methods) that have been widely used in previous studies to identify biomarkers^24,25^. For each dataset, we identified genes associated with prognosis and used these genes to construct the machine learning model. To check the accuracy of the predicted data, we used the C-index and AUC values to evaluate the performance of each model. An overview of the performance of the machine learning model for each dataset is shown in Fig. 3a. We found that most of the machine learning models achieved high C-index and AUC values (Fig. 3b,c), indicating the accuracy of these models. For example, we identified several exosomal genes that were significantly associated with ovarian cancer prognosis (Fig. 3d). The validation cohort of the GSE17260^49^ dataset was significantly divided into two different risk groups (Fig. 3e) with a 5-year AUC value up to 0.929 (Fig. 3f).

## Usage Notes

We collected and screened cancer-associated exosomal RNA molecules at multiple levels: Collecting experimental validation data and providing cancer-associated exosomal RNAs with high confidence based on published literature; Screening of cancer-related potential modifiers based on high-throughput bulk data using differential analysis and multiple feature carving of the dataset for data type, aiming to uncover more information on functional and immune infiltration differences between normal samples and patients; Based on single-cell sequencing data, analysis of the variation in expression of the candidate gene of interest in different states and the proportion of expression in different cell types to infer the relationship between this RNA and cancer development, and characterization of heterogeneity between patients and analysis of functional changes according to the pseudo time of cell development for the features of the dataset. Users can visit the Exmdb homepage (http://www.bio-server.cn/ExMdb) to explore the datasets.

## Data Availability

The source code used to generate the scientific content of the dataset is available on Github (https://github.com/HMU-BioServer/ExMdb).
